# 

**Published:** 1885-05

**Authors:** 


### EDITORIAL.

### The Societies' International Medical Congress.

The Ninth International Medical Congress will be held in Washington City in 1887. The following are some of the rules and regulations for that meeting:

1. The Congress will be composed of members of the regular medical profession, and of such persons as may be specially designated by the Executive Committee, who shall have inscribed their names on the register of the Congress, and shall have taken out their tickets of admission. As regards foreign members, the above conditions are the only ones which it seems at present expedient to impose. The American members of the Congress shall be appointed by the American Medical Association, by regularly organized State and local societies, and also by such general organizations relating to special departments and purposes, as the American Academy of Medicine, the American Surgical Association, the American Gynæcological Ophthalmological, Laryngological, Neurological and Dermatological Society, and the American Health Association. Each of the foregoing societies being entitled to appoint one delegate for every ten of their membership. Members of all special and subordinate committees appointed by the General Committee shall also be entitled to membership in the Congress. All societies entitled to representation are requested to elect their delegates at their last regular meeting, preceding the meeting of the Congress, and to furnish the Secretary General with a certified list of the delegates so appointed.

2. The work of the Congress is divided into nineteen sections, viz.:

1. Medical Education, Legislation, Registration, including methods of teaching and building, apparatus, etc., connected therewith.
2. Anatomy.
3. Physiology.
4. Pathology.
5. Medicine.
6. Surgery.
7. Obstetrics.
8. Gynæcology.
9. Ophthalmology.
10. Otology.
11. Dermatology and Syphilis.
12. Nervous Diseases. Phychiatry.
13. Laryngology.
14. Public and International Hygiene.
15. Collective Investigation, Nomenclature, and Vita Statistics.
16. Military and Naval Surgery and Medicine.
17. Practical and Experimental Therapuetics.
18. Diseases of Children.
19. Dental and Oral Surgery.

3. The general meetings will be reserved for the transaction of the business of the Congress, and for addresses or communications of scientific interest, more general than those given in the Sections.

4. Questions which have been agreed upon for discussion in the Section shall be introduced by members previously nominated by officers of the Section. Members who open discussions shall present in advance a statement of the conclusions which they have formed as a basis for debate.

5. Notices of papers to be read in any of the Sections, together with the abstracts of the same, must be sent to the Secretary of that Section before April 30th, 1887. These abstracts will be regarded as confidential communications, and will not be published until the meeting of the Congress. * * * *

6. All addresses or papers read at either meeting, or in the Sessions, are to be immediately handed to the Congress. The Executive Committee, after the conclusion of the Congress, shall proceed with the publication of the transactions, and shall have full power to decide which paper shall be published, whether in whole or in part.

7. The official languages are English, French, and German.

8. The rules, programmes, and abstracts of papers, will be published in English, French, and German. * * * * * * * * *

9. Officers of the General Committee on organization are—President, and such number of Vice-Presidents as may be hereafter determined upon; a Secretary General, and a Treasurer, and those elected to these positions will be nominated by the General Committee to hold the same offices in the Congress. All vacancies in those offices shall be filled by election. Honorary Presidents of the Congress, and of the several Sections, may be appointed at the meeting of the Congress.

10. There shall be an Executive Committee, to be composed of President, Secretary General, and Treasurer of the General Committee. * * * * * * * * *

11. There shall be a standing Committee on Finance, composed of such a number of persons as the Executive Committee may deem expedient, to be appointed by the President, subject to the approval of the Executive Committee. The Chairman of the Finance Committee shall be ex-officio one, of the Vice-president of the Congress, and also a member of the General Executive Committees. The Treasurer will be an ex-officio member of the Finance Committee.

12. Presidents of the Sections shall be ex-officio members of the General Committee.

13. The Committee on Organization of each Section shall be composed of a President, and such number of Vice-Presidents as may be deemed expedient, with one or more Secretaries and of members forming a council.

The organization of Section Nineteen, on Dental and Oral Surgery, is as follows:

### SECTION 19.—DENTAL AND ORAL SURGERY.

#### PRESIDENT.

Jonathan Taft, M. D., Cincinnati.

#### VICE-PRESIDENTS.

W. W. Allport, M. D., Chicago.
William H. Durinelle, M. D., New York.
Jacob L. Williams, M. D., Boston.

#### SECRETARY.

Edward A. Bogue, M. D., New York,

#### COUNCIL.

W. C. Barrett, M. D., Buffalo.
Thomas Fillebroun, M. D., Boston.
F. J. S. Gorgas, M. D., Baltimore.
Edward Maynard, M. D., Washington.
H. J. McKellops, D. D. S., St. Louis.
C. Newlin Peirce, D. D. S., Philadelphia.
L. D. Shepard, D. D. S., Boston.
James Truman, D. D. S., Philadelphia.
J. W. White, M. D., Philadelphia.

### Northern Ohio Dental Association.

The twenty-sixth annual meeting of this Society will be held in Youngstown, O., Wednesday, May 12th and 13th, commencing at 10 o'clock A. M. on Tuesday. The following programme is presented for papers and discussion.

1. Dental Hygiene.
2. Treatment of Diseased Teeth, Gums, and Pulps.
3. Pathology of Alveolar Abcesses.
   Paper by W. H. Whitslar, D. D. S.
4. Effects of Zymotic Disease upon Teeth.
5. Miscellaneous subjects.

This is a very active Association, and should call out a large number of the profession of Northern and Eastern Ohio, and indeed from elsewhere, so far as it is practicable to attend. Special effort has been made to have this meeting one of the best the Association has ever held, and it is to be hoped that the expectations will be fully realized.

### Mad River Dental Society.

The annual meeting of this Society will be held in the parlors of the Phillips House, Dayton, O., Tuesday, May 19, beginning at 10 o'clock and continuing one day only. The following programme of subjects is presented:

1. Dental Economics.
2. Different Materials for and Methods of Filling Teeth.
3. Treatment of Pyorrhoea Alveolaris.
4. Cases in Practice.

We hope that every member will be present.

THE Dental Society of the State of New York will hold its seventeenth annual meeting at Albany on Wednesday and Thursday, May 13th and 14th, 1885. The Board of Censors will meet on Tuesday, May 12, at 10 o'clock A. M., in Geological Hall, for the examination of credentials for the degree of "Master of Dental Surgery." Information as to the requirements of the Board may be obtained of the Secretary, Frank French, Rochester.

### CONTENTS.

### THE DENTAL REGISTER,

A MONTHLY MAGAZINE DEVOTED TO THE INTERESTS OF THE

**DENTAL PROFESSION.**

*Terms Reduced to* $2.00 *Per Year. Single Copies,* 25 *Cents.*

**EDITED BY PROF. J. TAFT, D.D.S.**

—AND—

**Published by SPENCER & CROCKER, Ohio Dental and Surgical Depot.**

The volume commences in January and closes in December.

The REGISTER being issued monthly is always fresh, therefore Indispensable to the practitioner who desires to keep posted up with the new inventions and improvements which are daily developed. Neither expense nor effort will be spared to give value and variety to its contents. Articles will be illustrated with well executed engravings whenever they will add to the interest or assist to explanation.

Its extensive circulation and frequency of issue make it one of the BEST DENTAL ADVERTISING MEDIUMS in the United States.

All subscriptions must begin with January or July.

Local or special notices, five lines or less, will be inserted for $3 each insertion; over five lines, 50 cts. per line.

All advertising bills payable in advance, or at furthest, after the issue of the first number containing the advertisement. All transient advertisements to be paid for in advance.

☞CONTRIBUTIONS SOLICITED.

Exclusively Editorial Communications should be addressed to the Editor.

The latest number of the REGISTER may be ordered with or without other goods at any time, and all letters should be addressed to

**SPENCER & CROCKER, Ohio Dental & Surgical Depot, Cincinnati, O.**

